# Correction: Retinal nerve fiber layer thickness predicts CSF amyloid/tau before cognitive decline

**DOI:** 10.1371/journal.pone.0236379

**Published:** 2020-07-14

**Authors:** Samuel Asanad, Michele Fantini, William Sultan, Marco Nassisi, Christian M. Felix, Jessica Wu, Rustum Karanjia, Fred N. Ross-Cisneros, Abhay P. Sagare, Berislav V. Zlokovic, Helena C. Chui, Janice M. Pogoda, Xianghong Arakaki, Alfred N. Fonteh, Alfredo A. Sadun, Michael G. Harrington

The fifteenth author's name is spelled incorrectly. The correct name is: Alfredo A. Sadun.

The following information is missing from the Funding statement: This study was also supported by Research to Prevent Blindness Inc. (unrestricted grant).

In the Discussion, there is an error in the third sentence of the second paragraph. The correct sentence is: Notably, this observed relationship between CSF protein levels and the retina is similar to that observed in the brain, where CSF Aß is inversely correlated with Aß load and neuropathology, while CSF Tau is directly correlated.

The figure legends in S1 Fig are incorrect. Please view the correct S1 Fig below.

Fig 1 is incorrect. The authors have provided a corrected version here.

**Fig 1:**
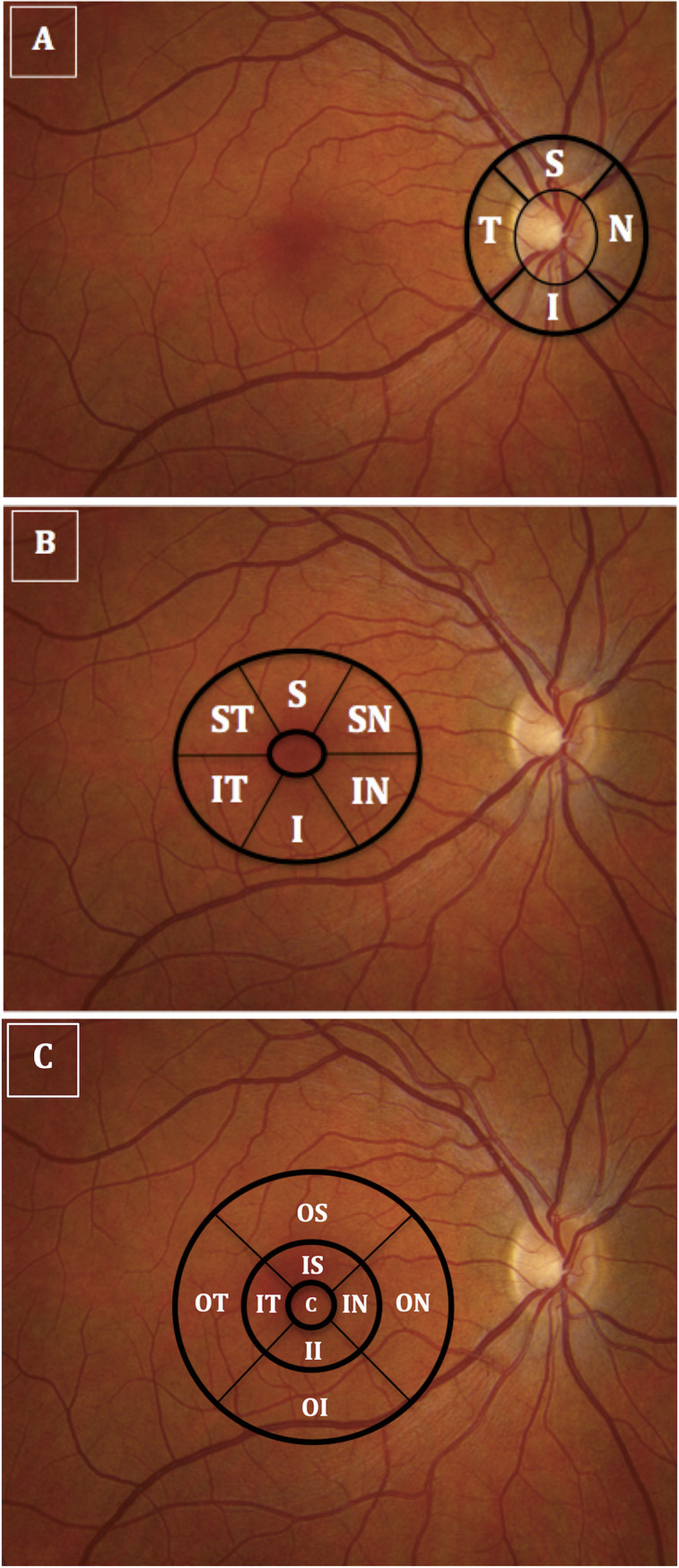
Illustrates optical coherence tomography imaging of the peripapillary and macular areas. (A) The thicknesses of the 4 retinal nerve fiber layer quadrants (temporal, superior, nasal, inferior); (B) ganglion cell-inner plexiform layer sectors (superior-temporal, superior, superior-nasal, inferior-nasal, inferior, inferior-temporal; (C) macular full-thickness sectors (center, inner-superior, outer-superior, inner-inferior, outer-inferior, inner-temporal, outer-temporal, inner-nasal, outer-nasal) were measured using peripapillary and macular circular scans centered on the disc and on the fovea, respectively).

## Supporting information

S1 Fig AD OCT scatter plots for OD: Series 1 = CH-PAT; Series 2 = CH-NAT. (PDF)

## References

[1] Asanad S, Fantini M, Sultan W, Nassisi M, Felix CM, Wu J, et al (2020) Retinal nerve fiber layer thickness predicts CSF amyloid/tau before cognitive decline. PLoS ONE 15(5): e0232785 10.1371/journal.pone.0232785 32469871 PMC7259639

