# Supplementary material for: Retinal nerve fiber layer thickness predicts CSF amyloid/tau before cognitive decline
Source: PLoS One. 2020 May 29;15(5):e0232785. doi: 10.1371/journal.pone.0232785 (PMC7259639; doi:10.1371/journal.pone.0232785)
Supplement: S1 Fig — (DOCX) [file pone.0232785.s001.docx]

**S1 Fig. AD OCT Scatter Plots for OD: Series 1 = CH-PAT; Series 2 = CH-NAT**

**RNFL**

**Macula**
