# Supplementary material for: Retinal nerve fiber layer thickness predicts CSF amyloid/tau before cognitive decline
Source: PLoS One. 2020 May 29;15(5):e0232785. doi: 10.1371/journal.pone.0232785 (PMC7259639; doi:10.1371/journal.pone.0232785)
Supplement: S1 Table — (DOCX) [file pone.0232785.s002.docx]

**S1 Table. Title for S1 Table.**

| **NAT** |  |  |  | **OD** |  |  |  |  | **OS** |  |  |  |  |
| --- | --- | --- | --- | --- | --- | --- | --- | --- | --- | --- | --- | --- | --- |
| **ID** | **A** | **T** | **A/T** | **S** | **I** | **T** | **N** | **Avg** | **S** | **I** | **T** | **N** | **Avg** |
| **1240** | **1025.59** | **292.19** | **3.51** | **114** | **123** | **55.5** | **94** | **94** | **114** | **120** | **48** | **94** | **94** |
| **1609** | **993.10** | **260.64** | **3.81** | **111.5** | **124.5** | **84.5** | **60.5** | **92.5** | **111.5** | **124.5** | **74** | **59** | **92.5** |
| **1369** | **1057.39** | **161.63** | **6.54** | **103.5** | **108.5** | **64.5** | **68.5** | **85.5** | **103.5** | **104** | **56** | **64.5** | **81.5** |
| **1423** | **637.46** | **138.59** | **4.60** | **98** | **118** | **50** | **70** | **83** | **98** | **101** | **50** | **61** | **78** |
| **1419** |  |  |  | **84** | **99** | **52** | **59** | **74** | **100** | **102** | **46** | **65** | **78** |
| **1440** | **817.31** | **181.96** | **4.49** | **126.5** | **114** | **54.5** | **83.5** | **94** | **126.5** | **100** | **53.5** | **71** | **87.5** |
| **1492** | **511.30** | **134.58** | **3.80** | **68** | **104** | **69.5** | **64.5** | **76.5** | **69** | **105.5** | **54** | **68.5** | **74** |
| **1349** | **1144.62** | **300.23** | **3.81** | **105.5** | **127** | **68.5** | **107.5** | **102** | **104** | **124.5** | **60.5** | **96** | **96.5** |
| **1449** | **756.60** | **155.05** | **4.88** | **135** | **121** | **66.5** | **89.5** | **102** | **135** | **121** | **62.5** | **89.5** | **102** |
| **1358** | **810.50** | **135.78** | **5.97** | **116.5** | **102** | **64** | **77** | **89** | **116.5** | **101** | **64** | **65.5** | **86.5** |
| **1325** | **818.74** | **122.01** | **6.71** | **110.5** | **127.5** | **60** | **99** | **95.5** | **105.5** | **127.5** | **58** | **84** | **93.5** |
| **1491** | **742.08** | **164.52** | **4.51** | **100** | **98.5** | **61.5** | **67.5** | **90** | **99.5** | **98.5** | **58** | **63.5** | **80** |
| **1476** | **620.03** | **142.30** | **4.36** | **79** | **103** | **72.5** | **71** | **84.5** | **90.5** | **104.5** | **72.5** | **70** | **84.5** |
| **1364** | **835.19** | **174.93** | **4.77** | **117** | **102** | **87** | **61.5** | **86.5** | **116** | **98.5** | **87** | **59** | **90.5** |
| **1414** | **1567.65** | **570.15** | **2.75** | **106.5** | **121** | **48.5** | **91.5** | **89** | **106.5** | **115.5** | **48.5** | **64** | **84** |
| **1256** | **587.32** | **426.07** | **1.38** | **91** | **102** | **57** | **62** | **79** | **99** | **112** | **63** | **54** | **82** |
| **1379** | **626.50** | **220.44** | **2.84** | **111** | **110** | **71** | **75** | **92** | **95** | **119** | **70** | **74** | **90** |
