# Supplementary material for: Retinal nerve fiber layer thickness predicts CSF amyloid/tau before cognitive decline
Source: PLoS One. 2020 May 29;15(5):e0232785. doi: 10.1371/journal.pone.0232785 (PMC7259639; doi:10.1371/journal.pone.0232785)
Supplement: S2 Table — (DOCX) [file pone.0232785.s003.docx]

**S2 Table. Title for S2 Table.**

| **PAT** |  |  |  | **OD** |  |  |  |  | **OS** |  |  |  |  | **Notes** |
| --- | --- | --- | --- | --- | --- | --- | --- | --- | --- | --- | --- | --- | --- | --- |
| **ID** | **A** | **T** | **A/T** | **S** | **I** | **T** | **N** | **Avg** | **S** | **I** | **T** | **N** | **Avg** |  |
| **1487** | **884.06** | **495.26** | **1.79** | **107** | **123** | **78** | **70** | **94** |  |  |  |  |  | OS dense cataract |
| **1311** | **666.49** | **279.94** | **2.38** | **108.5** | **122.5** | **51.5** | **74.5** | **92** | **122.5** | **122.5** | **57** | **74.5** | **94.5** |  |
| **1360** | **1210.88** | **481.32** | **2.52** | **95** | **123** | **59** | **84** | **92** | **98.5** | **123** | **59** | **87.5** | **92** |  |
| **1571** | **935.14** | **428.75** | **2.18** | **103** | **128** | **86** | **89** | **101** |  |  |  |  |  | OS retinal pathology |
| **1477** | **385.46** | **190.85** | **2.02** | **103.5** | **81.5** | **44.5** | **47.5** | **73** | **103.5** | **96.5** | **47.5** | **47.5** | **74** |  |
| **1646** | **2128.67** | **1757.01** | **1.21** | **93** | **115** | **59** | **75** | **86** | **93** | **115** | **63** | **75** | **86** |  |
| **1651** | **1364.87** | **597.33** | **2.28** | **96** | **94** | **61** | **56** | **77** | **103** | **86** | **56** | **64** | **77** |  |
| **1619** | **1401.91** | **1359.70** | **1.03** | **93** | **92** | **62** | **56** | **78** | **93** | **92** | **73** | **56** | **78** |  |
| **1657** | **984.12** | **396.81** | **2.48** | **99** | **122** | **59** | **59** | **85** | **93** | **124** | **61** | **66** | **86** |  |
| **1623** | **1111.16** | **2259.33** | **0.49** | **82** | **86** | **54** | **53** | **69** | **86** | **89** | **54** | **58** | **72** |  |
| **1649** | **1396.96** | **1145.90** | **1.22** | **90** | **102** | **74** | **70** | **85** | **93** | **102** | **90** | **77** | **90** |  |
| **1618** | **1570.66** | **1106.84** | **1.42** | **97** | **114** | **58.5** | **73.5** | **86.5** | **97** | **114** | **61.5** | **73.5** | **86.5** |  |
| **1268** | **815.79** | **870.68** | **0.94** | **131** | **125** | **51** | **72** | **97** | **131** | **133** | **51** | **72** | **97** |  |
| **1352** | **511.73** | **302.39** | **1.69** | **98** | **125** | **61** | **64** | **87** | **106** | **127** | **58** | **61** | **88** |  |
| **1617** | **786.07** | **921.05** | **0.85** | **91** | **93** | **54** | **65** | **78** | **102** | **100** | **54** | **65** | **80** |  |
| **1658** | **843.53** | **318.81** | **2.65** | **123** | **120** | **55** | **93** | **98** | **105** | **130** | **63** | **83** | **95** |  |
| **1634** | **1422.41** | **1160.94** | **1.23** | **96.5** | **98** | **72** | **58.5** | **83.5** | **96.5** | **105.5** | **84** | **58.5** | **86** |  |
| **1625** | **1040.78** | **1224.52** | **0.85** | **100** | **122.5** | **50.5** | **81** | **88.5** | **111.5** | **122.5** | **52** | **87.5** | **93** |  |
| **1425** | **709.05** | **707.43** | **1.00** | **86.5** | **89.5** | **53** | **65** | **75** | **92** | **89.5** | **53** | **65** | **75** |  |
| **1263** | **1153.28** | **1449.03** | **0.80** | **108** | **115** | **60** | **76** | **90** | **108** | **115** | **60** | **76** | **90** |  |
| **1647** | **1278.39** | **758.80** | **1.68** |  |  |  |  |  |  |  |  |  |  | RNFL data missing, will call back |
| **1327** | **792.53** | **706.25** | **1.12** | **102** | **125.5** | **53** | **67.5** | **88.5** | **108** | **125.5** | **53** | **67.5** | **88.5** |  |
| **1331** | **468.71** | **272.65** | **1.72** | **89** | **93** | **52.5** | **68.5** | **76** | **89** | **93** | **52.5** | **68.5** | **76** |  |
| **1628** | **1979.25** | **1234.98** | **1.60** | **102.5** | **104** | **51.5** | **74.5** | **84.5** | **111.5** | **104** | **52** | **75.5** | **85.5** |  |
| **1627** | **1766.87** | **1040.42** | **1.70** | **87** | **69.5** | **41** | **68** | **66** | **87** | **69.5** | **41** | **68** | **66** |  |
| **1429** | **813.28** | **521.80** | **1.56** | **107** | **113.5** | **60.5** | **69.5** | **89** | **115.5** | **113.5** | **60.5** | **78.5** | **92** |  |
