# Supplementary material for: Retinal nerve fiber layer thickness predicts CSF amyloid/tau before cognitive decline
Source: PLoS One. 2020 May 29;15(5):e0232785. doi: 10.1371/journal.pone.0232785 (PMC7259639; doi:10.1371/journal.pone.0232785)
Supplement: Attachment — Submitted filename: Response to reviewers.docx [file pone.0232785.s004.docx]

**Reviewer 1:**

**Comment 1:**

“First, I cannot see the number of cases included in the abstract.”

*Authors reply*

*We thank the reviewer for this comment and have clarified the number of cases in the abstract.*

**Comment 2:**

“Second, the conclusion might be a very categorical statement: “Retinal data have sufficient sensitivity to predict which patients have CSF biomarkers of AD pathology before cognitive deficits are detectable”, as this is only one study with a small number of patients.”

*Authors reply*

*We appreciate this comment and agree that this may be viewed as a categorical statement. We modify the manuscript by qualifying our conclusion according to the findings specific to our study.*

**Comment 3:**

“In line 113, the abbreviation of RGC-IPL should be explained.”

*Authors reply*

*We thank the reviewer for this comment and have modified the manuscript accordingly:*

**Comment 4:**

“Some references have a point after the journals and other not. Please amend the references section.”

*Authors reply*

*We appreciate this comment and we have amended the* ***References*** *accordingly.*

**Reviewer 2:**

**Comment 1:**

“The results seems to be somewhat conflicting with many previous publications that point to macular assessment as being more sensitive in detecting neuronal changes in both AD and MCI patients. How do the authors justify these differences? This should be addressed in more depth in the discussion. Studies involving patients with MCI show changes in macular thickness, especially with ganglion cell complexes around the central 3 mm of the fovea, while RNFL was preserved in these patients (Almeida, et al. Alzheimers Dement 2019).”

*Authors reply*

*We appreciate this comment and agree with providing a more in-depth discussion of our findings relative to previous studies. In agreement with the reviewer, we acknowledge previous findings suggesting progressive retinal thinning from MCI to AD. As referenced by the reviewer, Almeida et al. found ganglion cell complex thinning. We would like to explain that retinal thickness findings in MCI have been controversial. We add to the manuscript by describing previous studies conducted by Ascaso et al. and Oktem et al., which showed significant RNFL thinning in MCI and this thinning was found to significantly correlate with Mini-Mental State Examination (MMSE) scores (Ascaso et al., 2014; Oktem et al., 2015). We also reference studies, which showed no significant retinal thinning in MCI (Pillai et al., 2016; Feke et al., 2015). We further revise the manuscript by describing potential confounding factors that reportedly contribute to conflicting findings between studies including variability in OCT modalities, diagnostic criteria for subject enrollment, as well as the heterogeneity of MCI.* *We have modified the manuscript* ***Discussion*** *and have included the corresponding* ***References*** *accordingly.*

**Comment 2:**

“In these studies, it was also demonstrated that the thickness analysis divided into 9 sectors (and not six as performed in the present study) according to the ETDRS map seems to be more sensitive.”

*Authors reply*

*We appreciate and thank the reviewer for this comment. We would like to clarify that measurements for the GC-IPL were acquired using the standard 6-retinal sector elliptical annulus. With respect to macular full-thickness, measurements were determined using 9-retinal subfields, as defined by the Early Treatment Diabetic Retinopathy Study (ETDRS) as shown in the newly added, Figure 1C. This is consistent with previously published protocols using the spectral-domain OCT (Cirrus, Zeiss). As referenced by the reviewer, Almeida et al. and Cunha et al. similarly measured macular thickness using the ETDRS grid. We would like to mention that Santos et al. measured both ganglion cell complex macular full-thickness using a 4-sector grid. In addition, Almeida et al., Cunha et al., and Santos et al., each differ in their OCT technology and device models (SS-OCT, fd-OCT, and Heidelberg SPECTRALIS SD-OCT, respectively), which intrinsically vary in computational analysis. As suggested by the reviewer, we acknowledge that differences in OCT devices and segmentation algorithms may contribute to variable retinal thickness findings.* *We have modified the manuscript* ***Methods*** *and* ***Discussion*** *sections to reflect these changes.*

**Comment 3:**

“These similar findings were also demonstrated in patients with AD in other studies (Santos CY, et al. Alzheimers Dement 2018 and Cunha LP, et al. PLoS One 2016). There is sufficient evidence suggesting that there is a primary involvement of the retina in patients with AD and that these changes (neuronal impairment with decreased ganglion cell complex) may occur earlier than axonal loss, which contrasts with the findings of the present study.”

*Authors reply*

*We appreciate this comment and agree with further discussing our findings in relation to previous studies in AD. As suggested by the reviewer, we justify our findings by describing previous studies in AD, which have similarly* shown RNFL thinning and a significant correlation to visual function and cognitive deficits^43-52^. We would like to mention that the referenced AD study conducted by Cunha et al. study showed both ganglion cell complex and RNFL axonal loss; and these were both shown to significantly correlate with cognitive function testing. In addition, studies conducted by Martin et al. on retinal thickness changes in relation to AD duration and severity showed RNFL thinning preceded ganglion cell loss. We further support our results by discussing recent findings reported by our laboratory in AD using postmortem histopathology^54^. We would also like to point out that Santos et al. primarily found RNFL axonal loss in preclinical AD^57^. Ganglion cell layer, inner plexiform layer, as well as outer nuclear layer thickness changes were regarded as non-significant findings. *We have modified the manuscript* ***Discussion*** *and the corresponding* ***References*** *to reflect these changes.*
