# Supplementary material for: Correction: Retinal nerve fiber layer thickness predicts CSF amyloid/tau before cognitive decline
Source: PLoS One. 2020 Jul 14;15(7):e0236379. doi: 10.1371/journal.pone.0236379 (PMC7360049; doi:10.1371/journal.pone.0236379)
Supplement: S1 Fig — (PDF) [file pone.0236379.s001.pdf]

AD OCT Scatter Plots for OD: CH-NAT vs CH-PAT

RNFL

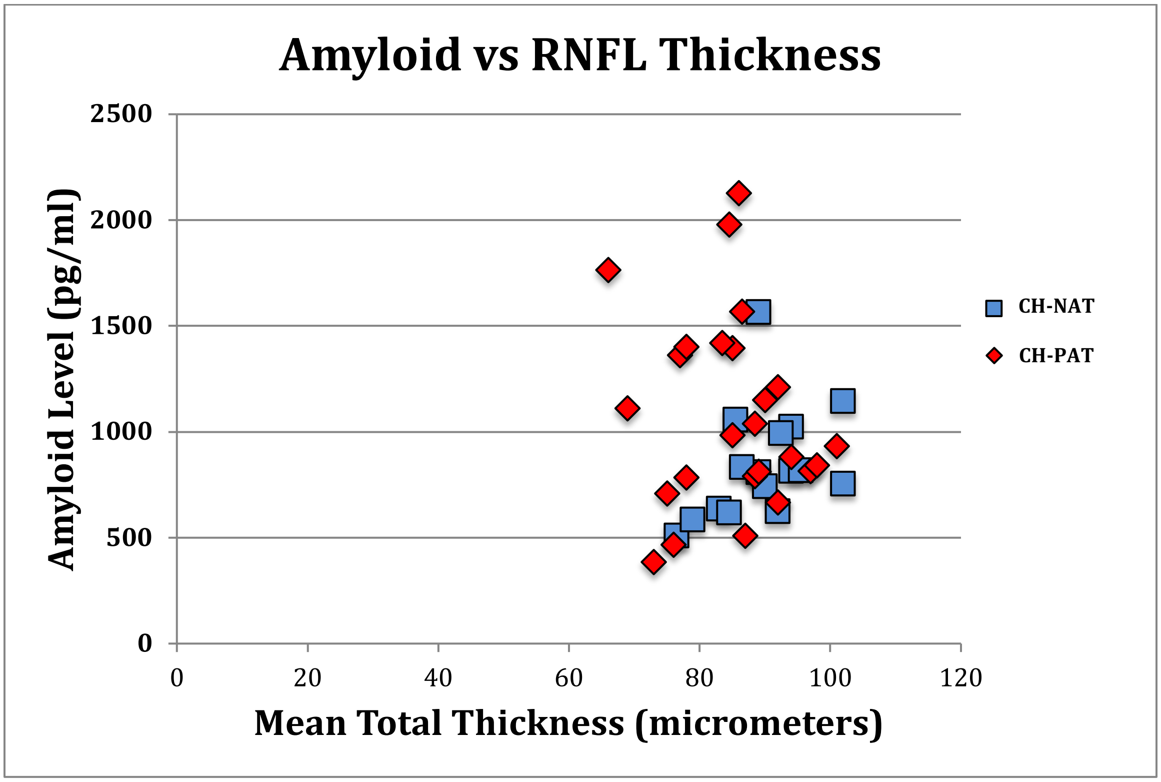

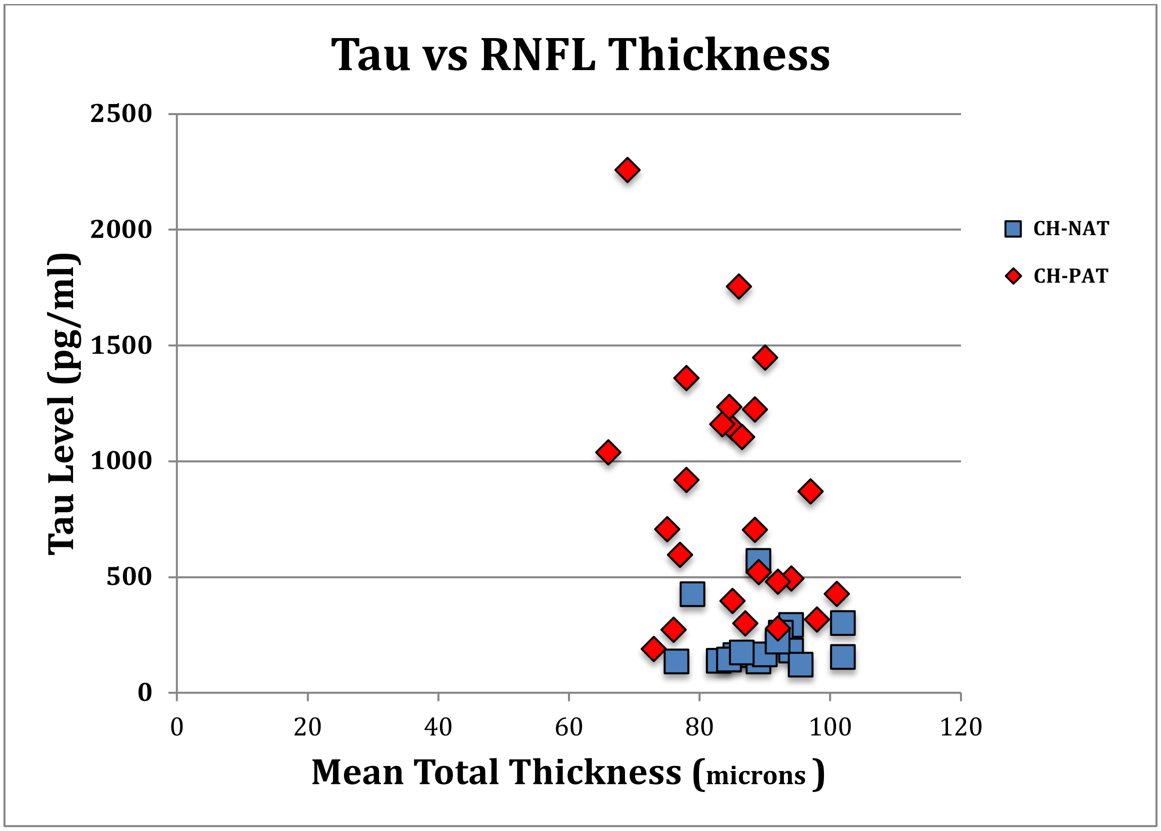

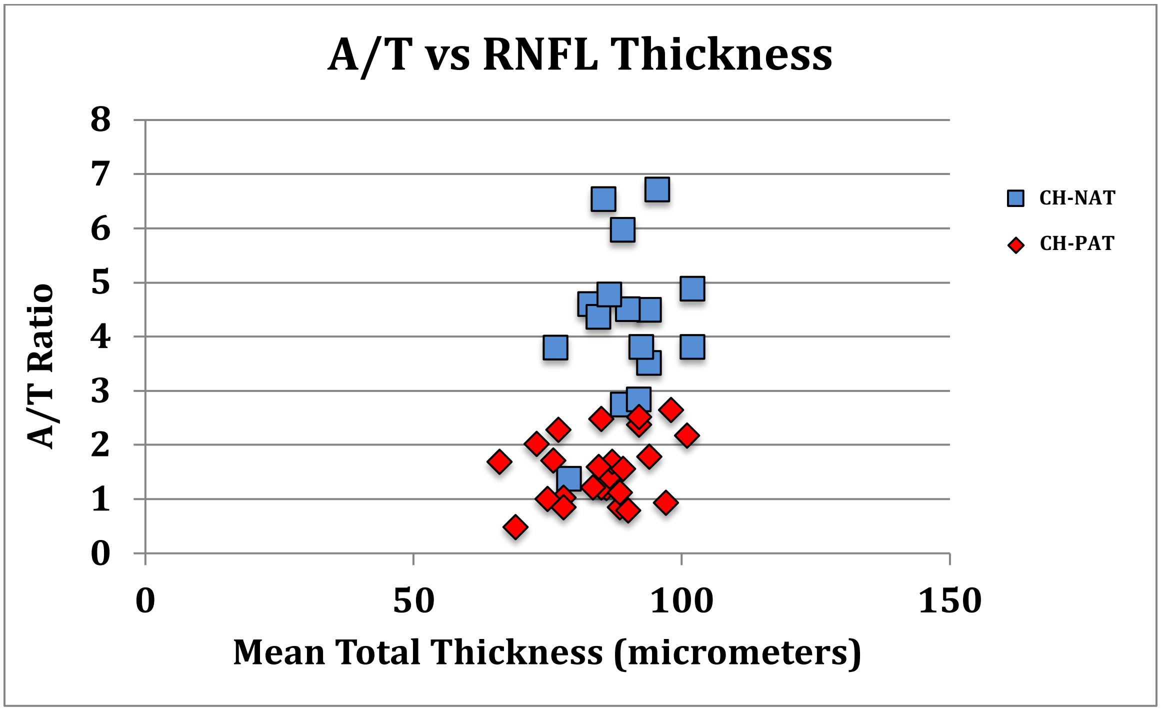
A/T vs RNFL Thickness
A/T Ratio
8
7
6
5
4
3
2
1
0
0
50
100
150
Mean Total Thickness (micrometers)
CH-NAT
CH-PAT

GCC

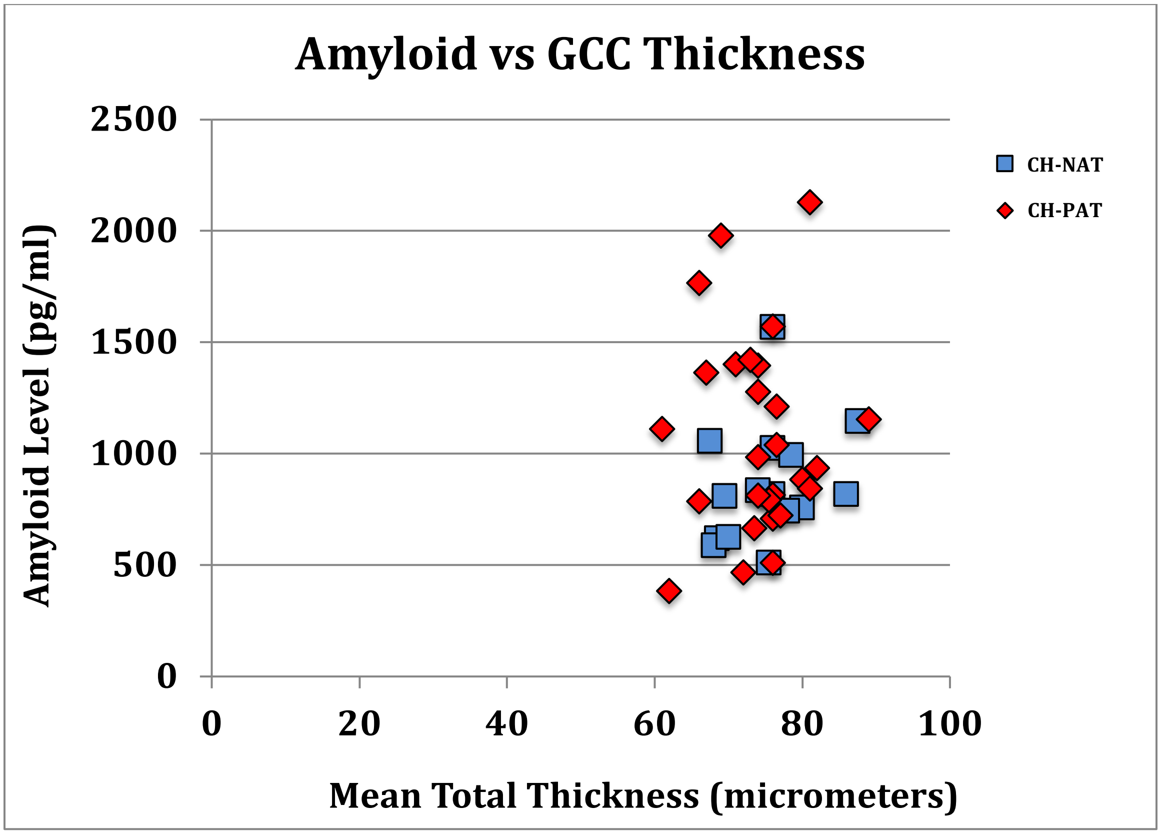
Amyloid vs GCC Thickness
Amyloid Level (pg/ml)
2500
2000
1500
1000
500
0
0
20
40
60
80
100
Mean Total Thickness (micrometers)
CH-NAT
CH-PAT

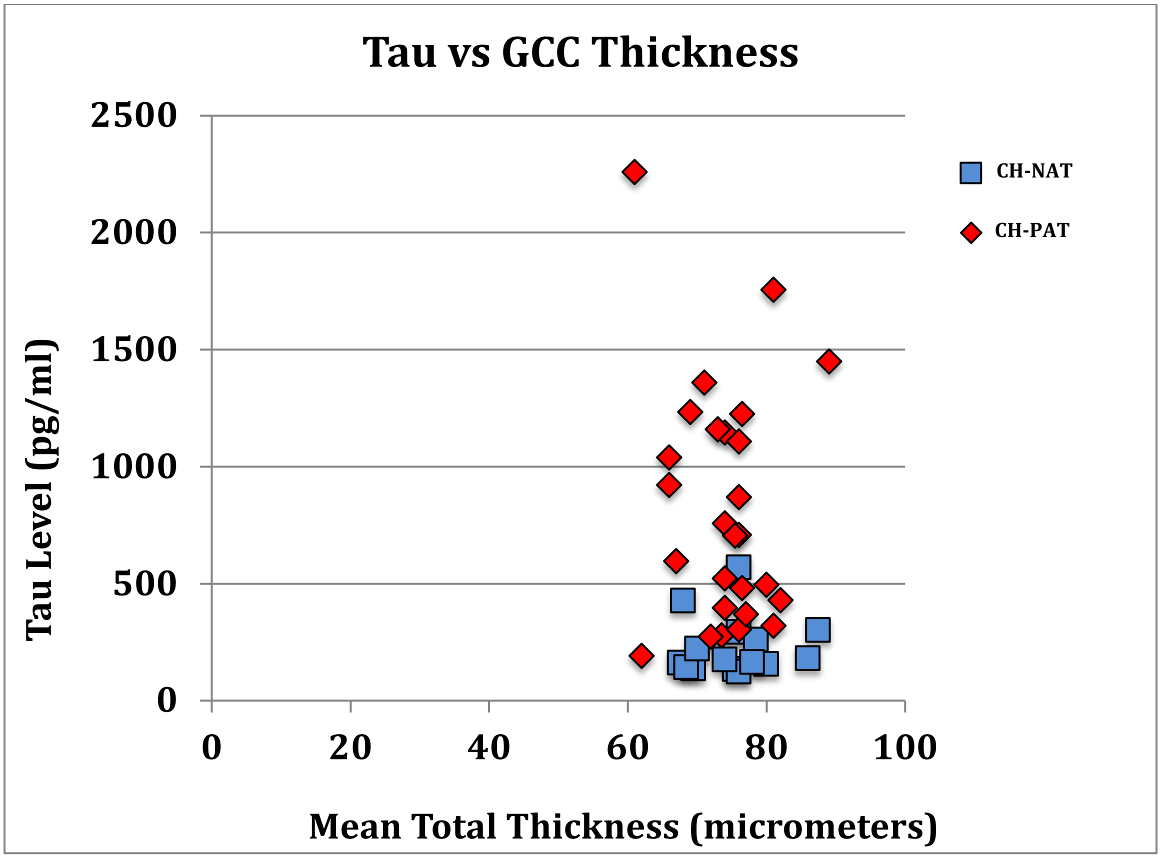
Tau vs GCC Thickness
Tau Level (pg/ml)
2500
2000
1500
1000
500
0
0
20
40
60
80
100
Mean Total Thickness (micrometers)
CH-NAT
CH-PAT

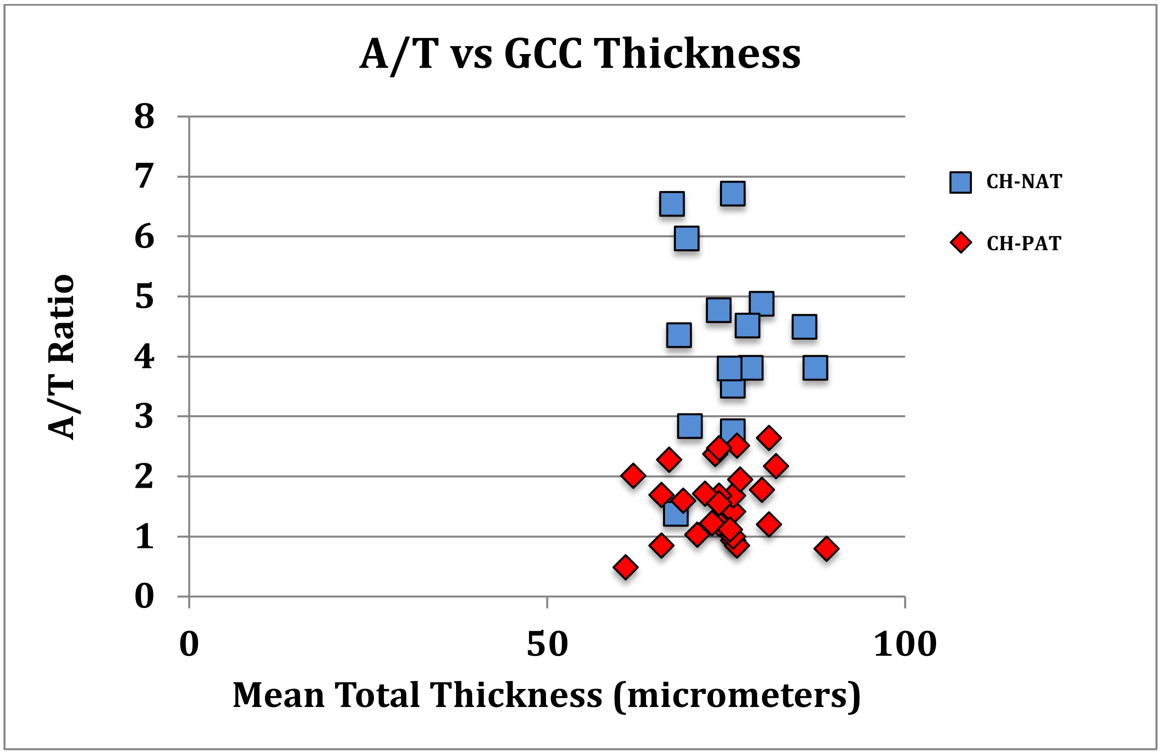
A/T vs GCC Thickness
A/T Ratio
8
7
6
5
4
3
2
1
0
0
50
100
Mean Total Thickness (micrometers)
CH-NAT
CH-PAT

Macula

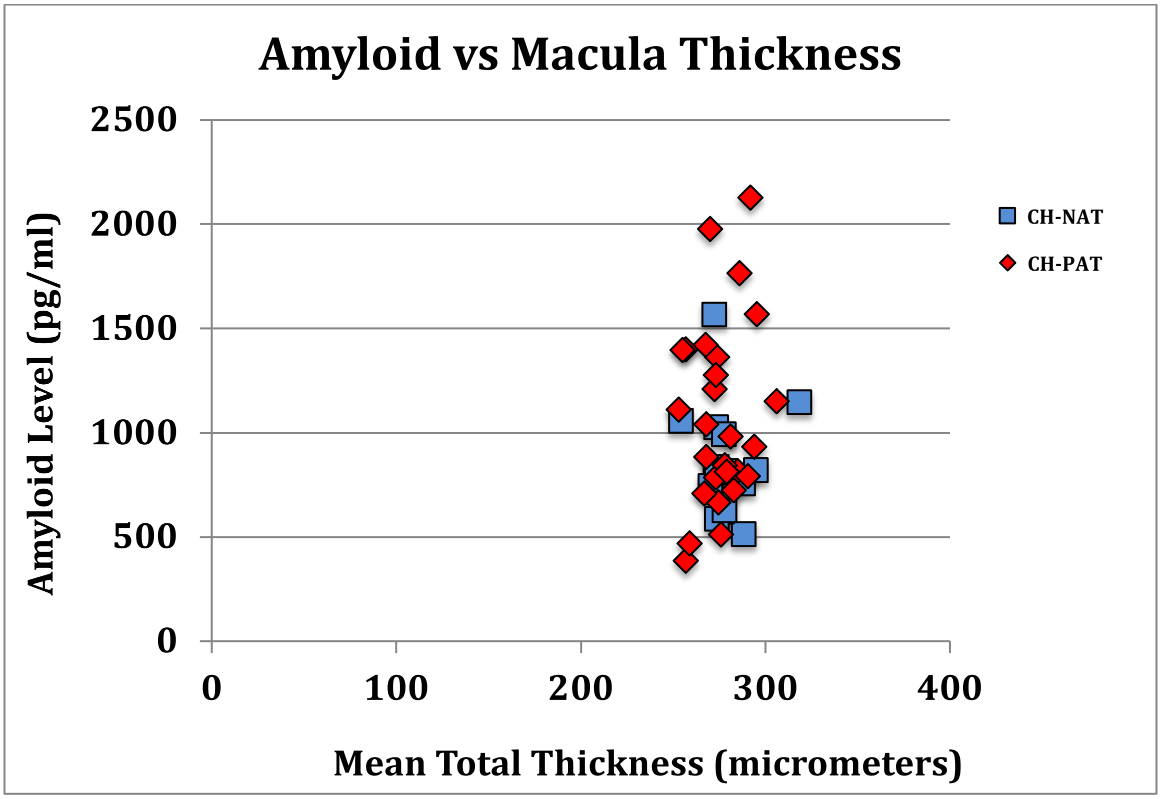
Amyloid vs Macula Thickness
2500
2000
1500
1000
500
0
0
100
200
300
400
Amyloid Level (pg/ml)
Mean Total Thickness (micrometers)
CH-NAT
CH-PAT

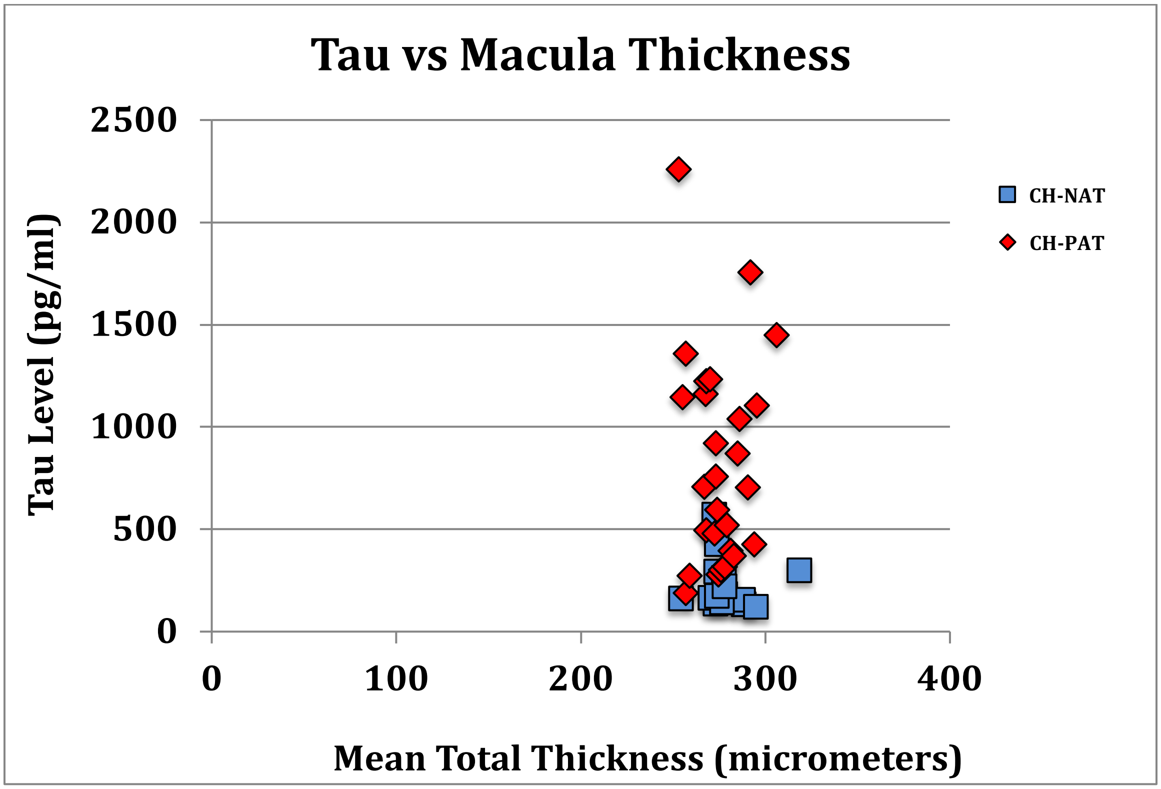
Tau vs Macula Thickness
2500
2000
1500
1000
500
0
0
100
200
300
400
Tau Level (pg/ml)
Mean Total Thickness (micrometers)
CH-NAT
CH-PAT

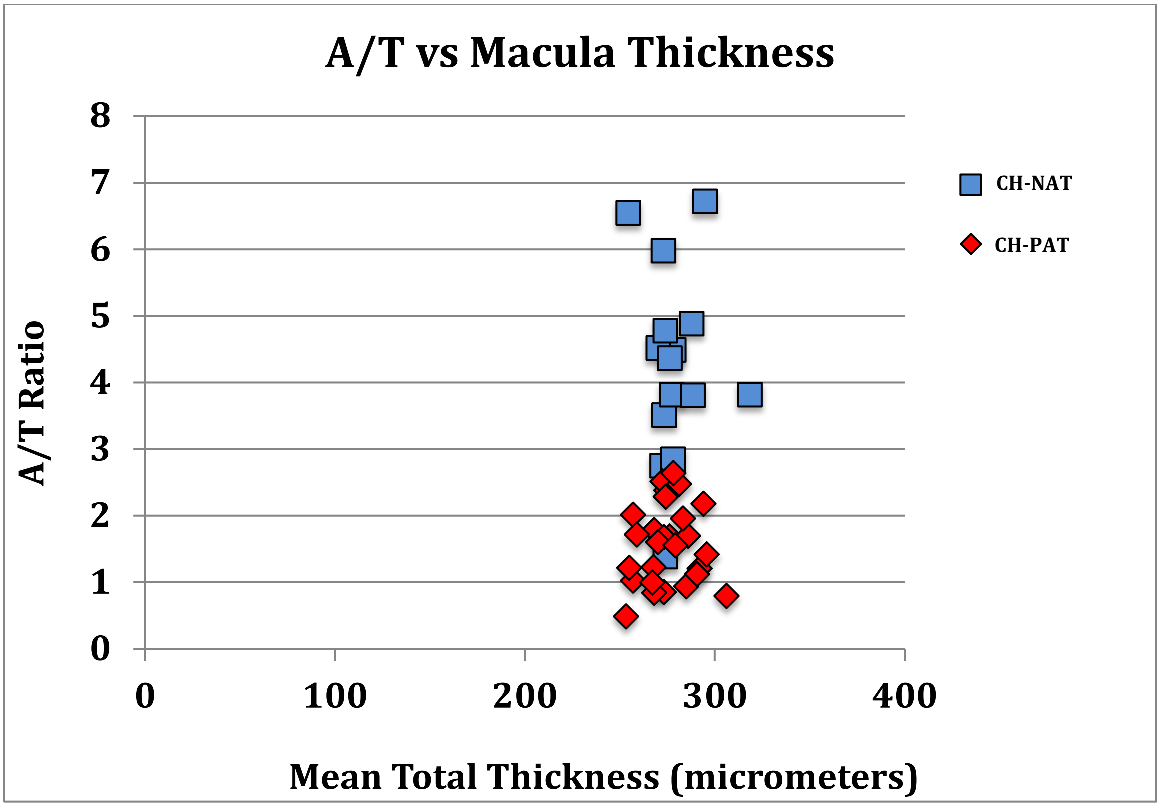

A/T vs Macula Thickness
A/T Ratio
Mean Total Thickness (micrometers)
0
1
2
3
4
5
6
7
8
0
100
200
300
400
CH-NAT
CH-PAT
